# Assessment of Mutation Drift Equilibrium and the Occurrence of a Recent Genetic Bottleneck in South Indian Zebu Cattle

**DOI:** 10.3390/ani12141838

**Published:** 2022-07-19

**Authors:** Vandana Manomohan, Saravanan Ramasamy, Rudolf Pichler, Murali Nagarajan, Sivakumar Karuppusamy, Sudhakar Krovvidi, Raja K. Nachiappan, Sunday O. Peters, Kathiravan Periasamy

**Affiliations:** 1 Animal Production and Health Section, Joint FAO/IAEA Division, International Atomic Energy Agency, 1400 Vienna, Austria; rudolf.pichler@iaea.org (R.P.); kathiravan.periasamy@fao.org (K.P.); 2 Veterinary College and Research Institute, Tamil Nadu Veterinary and Animal Sciences University, Chennai 600051, India; murali.vete@gmail.com (M.N.); lpmsiva.69@gmail.com (S.K.); 3 NTR College of Veterinary Science, Sri Venkateswara Veterinary University, Tirupati 521101, India; vetsreesudha@rediffmail.com; 4 National Bureau of Animal Genetic Resources, Karnal 132001, India; drknraja@yahoo.co.in; 5 Department of Animal Science, Berry College, Mount Berry, GA 30149, USA; speters@berry.edu; 6 Animal Genetics Resources Branch, Animal Production and Health Division, Food and Agriculture Organization of the United Nations, 00100 Rome, Italy

**Keywords:** zebu, microsatellite, mutation models, heterozygosity excess, mode shift

## Abstract

**Simple Summary:**

This study was conducted on eight Indian zebu breeds with an objective to reveal any cryptic genetic bottlenecks in the population. The alarming reduction in the effective population size of these breeds was the major reason for conducting this study. Genotyping of 27 FAO/ISAG-recommended microsatellite markers and further statistical analysis using allele frequency data under three different models of mutation revealed the absence of recent genetic bottlenecks in any of the populations under study. The results from the qualitative test of mode shift distortion were in accordance with the above findings. Even though the results of the present study indicated little or no critical loss of rare alleles in any of the breeds understudy, the chances of this happening cannot be completely ignored. One of the plausible explanations for this scenario could be the potential gene flow from commercial taurine cattle through indiscriminate crossbreeding in the native tract of these breeds. Thus, we here emphasize the need of further initiatives for improving selective breeding practices in order to conserve and effectively utilize the existing South Indian zebu cattle genetic diversity.

**Abstract:**

During the last few decades, the effective population size of indigenous zebu cattle breeds has declined drastically, resulting in the classification of some of them into the vulnerable, endangered, or critically endangered category. Drastic reductions in the effective size of a population may result in genetic bottlenecks and can affect within-breed genetic variability and its viability. The present study was undertaken with the objective of evaluating South Indian zebu cattle populations for mutation drift equilibrium and to detect the occurrence of recent genetic bottleneck events. A total of 293 cattle from eight indigenous breeds were genotyped at 27 FAO/ISAG-recommended microsatellite marker loci. Three different statistical tests, viz., the sign test, standardized differences test, and Wilcoxon sign rank test were performed using allele frequency data to detect loci with heterozygosity excess under the infinite alleles, stepwise, and two-phase mutation models. Under the infinite alleles model, the observed number of loci with heterozygosity excess (He > Heq) ranged between 10 and 19 among the investigated cattle breeds. However, the observed heterozygosity excess was not statistically significant (*p* > 0.05) in any of the studied breeds. Similarly, the standardized differences test and Wilcoxon sign rank test revealed no concrete evidence for the occurrence of a recent genetic bottleneck in South Indian zebu cattle breeds. The qualitative test for mode-shift distortion revealed a normal L-shaped distribution of allele frequencies, suggesting a lack of evidence for the loss of low-frequency alleles in all the investigated South Indian zebu cattle breeds.

## 1. Introduction

History depicts, with evidence, that the wild aurochs or *Bos primigenius* diverged into *Bos taurus* and *Bos indicus* between 280,000 [1] and 330,000 YBP [2] in the fertile crescent. Later, at around 8000 YBP, the domestication of *B. indicus* probably took place in the Indus valley region in modern-day Pakistan [3]. Over the years, due to long-term natural selection, these animals developed resilience to heat, humidity, poor-quality feedstuff, and various diseases of the tropics. India, being a mega diversity hot spot, harbors a diverse range of zebu cattle breeds. According to the second report on the state of the world’s animal genetic resources for food and agriculture—FAO—there are 14 registered breeds in Southern India, including two exceptionally dwarf breeds called Punganur and Vechur [4].

Even though India has the second largest cattle population in the world, the indigenous cattle population has shown a decline of six per cent during the last five years [5]. The major decline was observed in the population of male indigenous cattle (29.1 per cent), either due to the ban of rearing male animals for slaughter, the mechanization of agricultural activities, or the widespread acceptance of artificial insemination in cattle [5]. The shift in farming trends towards cattle with a high potential for milk production was clearly reflected in the 26.9% increase in the crossbred cattle population during the same period. Most of the cattle, especially the south Indian cattle, were draught or dual-purpose types that were used for ploughing or carting during earlier days. The drastic decline in the population size of indigenous South Indian cattle breeds has resulted in the classification of some of them into the vulnerable (e.g., Bargur), endangered (e.g., Pulikulam, Punganur), or critically endangered (e.g., Vechur) category [6]. 

Intensified efforts for conservation and the sustainable use of farm animal genetic resources are essential to prevent and reverse genetic diversity erosion. Drastic reductions in the effective size of a population may result in demographic bottlenecks and can affect within-breed genetic variability—particularly allelic diversity [7]. Reduced genetic diversity and increased inbreeding are bound to affect the viability of small populations [8] due to their inability to withstand extreme natural selection pressures. Genetic monitoring of demographic bottlenecks can help to detect any deviations from mutation drift equilibria and the occurrence of cryptic genetic bottlenecks in the population; this is done based on tests for heterozygosity excess at multiple microsatellite loci and have been employed successfully in various studies that reported genetic bottlenecks in livestock (e.g., Bargur cattle of south India [9]; Mehsana breed of Indian riverine buffalo [10]; goats [11], horses [12], reindeer [13], etc.). The present study was undertaken to evaluate the mutation drift equilibrium and to detect the cryptic genetic bottlenecks, if any, of eight south Indian draught and dual-type cattle breeds. 

## 2. Materials and Methods

A total of 293 blood samples from eight different indigenous cattle breeds, viz., Kangayam, Umblachery, Pulikulam, Deoni, Ongole, Hallikar, Vechur, and Punganur were utilized from their breeding tract for the present study. The photographs, distribution, geography, origin, and genetic relationships of the cattle breeds under investigation are available in a related publication from the authors [14]. The cattle breeds under study are maintained by small holders under a low input production system. Documented pedigree information is mostly unavailable under these management systems, but farmers are normally well aware of the breeding practices (including information on the bulls used for natural service or artificial insemination, calving, etc.). The sampling of cattle was performed as per the recommendations made in the FAO Guidelines [15]. Briefly, a stratified random sampling procedure was followed to collect blood from unrelated cattle with typical phenotypic features and located in randomly selected villages of the native breed tract. With the absence of pedigree records in most instances, unrelatedness was ensured by interviewing the farmers about the cattle’s breeding history. Furthermore, an inter-individual kinship matrix (KSC_Ind) based on the STR data was generated using Microsatellite Analyzer (MSA), and the back-transformed values (1-kf to kf) were utilized to confirm the unrelatedness of the sampled individuals.

Blood was collected by jugular venipuncture in EDTA-coated vacutainer vials. DNA was extracted following the standard phenol chloroform method [16]. The isolated genomic DNA was utilized for genotyping 27 FAO/ISAG-recommended microsatellite markers for cattle [15]. The forward primer for each marker was conjugated with one of the three fluorescent dyes (FAM, HEX, and ATTO) for multiplex capillary electrophoresis. The details of the annealing temperatures and polymerase chain reaction conditions are described elsewhere [17]. The allele size data for each sample were then extracted using GeneMapper v.4.1 software (Applied Biosystems, Waltham, MA, USA).

To identify any loci with heterozygosity excess, three tests, viz., the sign test, standardized differences test and Wilcoxon sign rank test were performed under the assumption of three different mutation models using allele frequency data [18]. The mutation models of microsatellite evolution followed in the study were the infinite alleles model (IAM), stepwise mutation model (SMM), and two-phase model (TPM). Furthermore, a qualitative test of mode shift was also done to detect whether the population has undergone any recent bottleneck using BOTTLENECK program [19].

## 3. Results

The basic diversity indices like mean observed number of alleles (ko), mean expected Hardy-Weinberg equilibrium (HWE) heterozygosity (He), and mean expected mutation drift equilibrium heterozygosity (Heq) under different mutation models are presented in Table 1.

Among the investigated cattle breeds, the allelic diversity was lowest in Vechur and Punganur cattle (ko = 6.04). Similarly, the mean expected HWE heterozygosity was lowest in Vechur cattle (He = 0.672).

The mean expected HWE heterozygosity (He) varied between 0.662 (Kangayam) and 0.741 (Punganur) among the studied cattle breeds. The mean expected mutation drift equilibrium heterozygosity (Heq) was estimated under the assumption of three models of microsatellite evolution, viz., the infinite allele model (IAM), stepwise mutation model (SMM) and two-phase model (TPM). The estimated Heq for the investigated breeds was lowest under the IAM with the mean values ranging from 0.619 (Kangayam) to 0.753 (Pulikulam), while it was highest under the SMM with the mean values varying between 0.725 (Kangayam) and 0.823 (Pulikulam). The estimated Heq under the TPM was intermediate between the IAM and SMM and ranged from 0.676 (Kangayam) to 0.792 (Pulikulam) among the investigated breeds. 

To evaluate the investigated cattle breeds for deviations from mutation drift equilibrium, three different statistical methods, viz., the sign test, standardized differences test, and Wilcoxon sign rank test were employed. All the three tests compared the mean expected HWE heterozygosity (He) and mean expected mutation drift equilibrium heterozygosity (Heq) under the assumption of the IAM, SMM, and TPM. Under the IAM, the observed number of loci with heterozygosity excess (He > Heq) ranged between 10 (Pulikulam) and 19 (Kangayam and Deoni) among the investigated cattle breeds (Table 2).

With the exception of the Pulikulam, the number of loci with heterozygosity excess (He > Heq) exceeded the number of loci with heterozygosity deficiency (Heq > He). However, the observed heterozygosity excess was not statistically significant in any of the studied breeds (*P* > 0.05). Under SMM, the number of loci with heterozygosity excess (He > Heq) varied from 4 (Deoni) to 10 (Umblachery), and the number of loci with heterozygosity deficiency (Heq > He) exceeded the number with heterozygosity excess in all the breeds investigated. Under the TPM, the observed number of loci with heterozygosity excess was intermediate between the IAM and SMM—thus ranging between 8 (Pulikulam) and 15 (Deoni). Under this mutation model, the observed number of loci with heterozygosity excess exceeded the number of loci with heterozygosity deficit only in the Hallikar breed, but without any statistical significance (*P* > 0.05). Thus, the sign test did not reveal significant deviations from the mutation drift equilibrium in any of the investigated South Indian draught cattle breeds. 

The results of the standardized differences test under different mutation models are presented in Table 3. The standardized differences test is a parametric test and takes into account the magnitude of heterozygosity excess/deficiency [20]. The T2 statistic of the standardized differences tests is calculated by dividing the difference between the HWE (He) and mutation drift equilibrium (Heq) heterozygosity with the standard deviation of the corresponding distributions of gene diversities. The calculated T2 statistic was further compared to an N (0, 1) distribution. Positive T2 statistics indicate heterozygosity excess, while negative T2 statistics indicate heterozygosity deficiency. Under the IAM, the T2 statistics were positive in five out of the eight studied breeds and ranged between 0.344 (Punganur) and 1.830 (Kangayam). Among these breeds, the T2 statistics were significant in the Kangayam cattle (*P* < 0.05) indicating a deviation from the mutation drift equilibrium. However, under the SMM, the T2 statistics were negative and statistically significant in all the breeds (*P* < 0.01). Similarly, under the TPM, the T2 statistics were negative in all the studied breeds. but significant in five of them—with the exception of the Kangayam, Hallikar, and Punganur. The results of the one-tailed Wilcoxon sign rank test for heterozygosity under different mutation models are presented in Table 4.

Under the IAM, the Deoni and Kangayam cattle breeds showed significant heterozygosity excess and deviations from the mutation drift (*P* < 0.05). However, such a heterozygosity excess was not observed in these populations when assumed under the SMM and TPM models. These differences in detection of significant heterozygosity excess across the mutation models have been reported earlier in Indian poultry [21], Marathwada buffaloes of Central India [22], and Indian buffalo populations [10].

**Figure 1:**
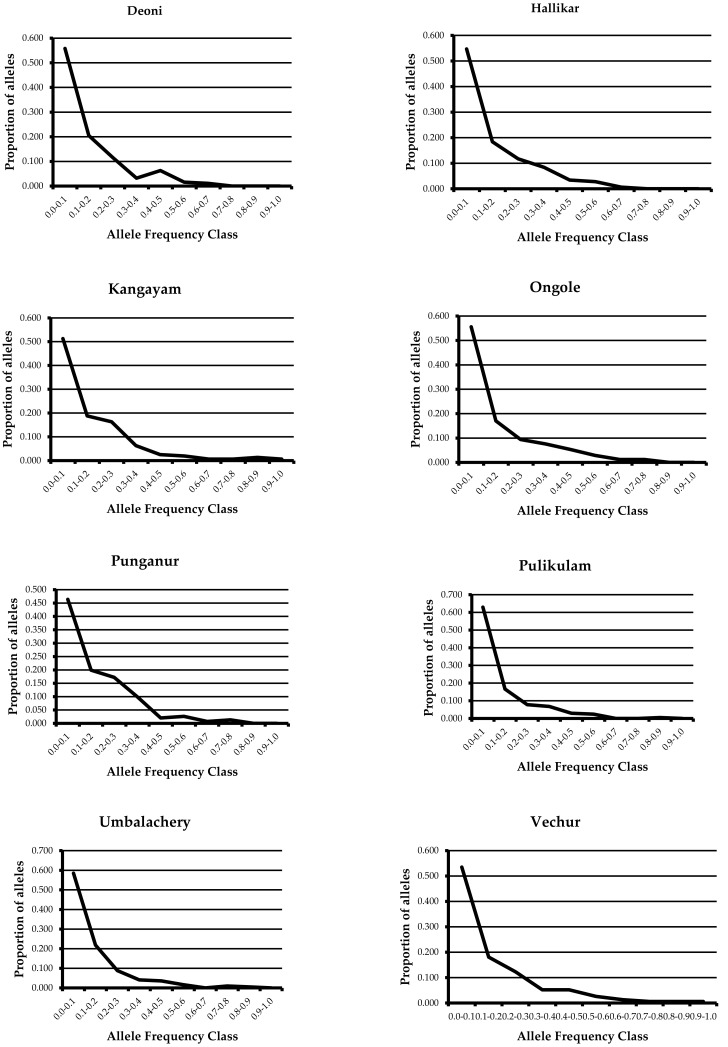
Qualitative test of mode shift for the detection of recent genetic bottlenecks in South Indian Zebu cattle breeds.

With genetic bottleneck events expected to result in the loss of rare alleles, a qualitative graphical test plotting the allele frequency distribution will show a characteristic mode-shift distortion from the normal L-shaped distribution. The graphical method consists of grouping alleles into each of 10 allele frequency classes and then plotting a frequency histogram. In the present study, all the eight cattle breeds revealed a normal L-shaped distribution of allele frequencies (Figure 1), suggesting a lack of evidence for the loss of low-frequency alleles.

## 4. Discussion

The allelic diversity was lowest in the Vechur and Punganur breeds of cattle and the mean expected HWE heterozygosity was lowest in the Vechur. The population size of the two dwarf cattle breeds has steadily declined in the past few decades. As per the DADF [23], the total population size of Vechur and Punganur cattle was 1065 and 2772, respectively, with anadult female population of 494 and 1077, respectively. The estimates of the adjusted effective population size for these breeds were 3 and 201, respectively [24], raising concerns of significant inbreeding. Recent reductions in effective population sizes can cause a correlative reduction in the observed number of alleles and gene diversity. At highly polymorphic short tandem repeat marker loci, low frequent alleles are expected to be lost quickly. Thus, the relatively low levels of allelic diversity in the Vechur and Punganur breeds among the investigated cattle could be due to small population sizes and high rates of inbreeding.

The purpose of this study was to evaluate indigenous South Indian cattle breeds for the occurrence of recent genetic bottlenecks. Most of these cattle breeds are draught or dual-purpose types that were used for ploughing or carting during earlier days. During the last few decades, due to mechanization and other reasons, the population size of many of these breeds has declined drastically. For example, only few hundred breedable females and <5 breedable males are available for the Vechur breed, with an estimated effective population size of <10. Punganur cattle have a total population of less than 3000 and an effective population size of ~200. Similarly, the effective population size of the Umblachery and Bargur breeds is 1580 and 1581, respectively [24]. Similarly, the effective population size of other South Indian cattle breeds are declining at a faster rate—mainly due to the lack of availability of purebred bulls and the increasing preference of farmers for crossbred cattle. This has resulted in the classification of South Indian breeds into the vulnerable (e.g., Bargur), endangered (e.g., Pulikulam, Punganur), or critically endangered (e.g., Vechur) category. Drastic reductions in the effective size of a population may result in demographic bottlenecks and can affect within-breed genetic variability—particularly allelic diversity. Reduced genetic diversity and increased inbreeding are bound to affect the viability of small populations due to their inability to withstand extreme natural selection pressures. 

The estimated Heq is consistent with earlier studies that have reported calculations of the mean expected mutation drift equilibrium heterozygosity under the assumption of different mutation models [20]. The IAM assumes that the mutation at a microsatellite locus can result in the insertion or deletion of any number of tandem repeat units, leading to a new allele state that did not previously exist in the population. In contrast, the SMM assumes that the mutation results in the addition or deletion of a single repeat unit at a locus—thus implying that two alleles differing by one repeat are more closely related than alleles that differ by many repeat units. Hence, for any given data set, the IAM predicts a lower mutation drift equilibrium heterozygosity than the SMM. Both the IAM and SMM represent extreme models of mutation, and it is not appropriate to assume that a microsatellite locus has evolved using either one of these models over the evolutionary scale of time. Hence, a two-phase model was proposed by Di Rienzoet al. [25] that assumes that a microsatellite locus evolves in the IAM and SMM at varying proportions over time. In the present study, the variance of the two-phase model was assumed to be 70% one-step mutations and 30% multistep/infinite changes.

The deviation of a population from the mutation drift equilibrium was tested here with three parametric tests.viz., the sign test, standardized differences test, and Wilcoxon sign rank test [18]. All the three tests compared the mean expected HWE heterozygosity (He) and mean expected mutation drift equilibrium heterozygosity (Heq) under the assumptions of the IAM, SMM, and TPM. None of the breeds showed significant deviations from the mutation drift equilibrium under all three models of mutation. Recently, bottlenecked populations have been expected to have lost rare alleles, but may still retain some heterozygosity that will be lost more slowly compared to allelic variation. All the above statistical tests were aimed at detecting this genetic signature of transient excess of heterozygosity. For selectively neutral loci such as microsatellites, allele number and frequency distribution in a natural population result from the dynamic equilibrium between mutation and genetic-drift. Non-bottlenecked populations that are near the mutation-drift equilibrium for selectively neutral loci are expected to have a large proportion of alleles at a low frequency. Such low-frequency alleles are expected to be more abundant than alleles at an intermediate frequency, regardless of the mutation rate and model [26].

Furthermore, the qualitative test plotting the allele frequency distribution was carried out in all the breeds. This will show a characteristic mode-shift distortion from the normal L-shaped distribution if the population has undergone a genetic bottleneck. The graphical method consists of grouping alleles into each of 10 allele frequency classes and then plotting a frequency histogram. However, none of the breeds showed a distortion of the L-shaped graph, depicting the absence of genetic bottlenecks in all the breeds studied. 

The detection of bottlenecks is complicated by several factors, including the timing and duration of the bottleneck, the extent of decline in population size, immigration, and the level of pre-bottleneck genetic diversity. All these factors can potentially obscure the genetic signals of population declines [20,27,28]. Girod et al. [29] and Peery et al. [30] showed the limited power of heterozygosity excess-based bottleneck tests in detecting declines in effective population size—particularly when the severity of bottleneck is low. There is an apparent decline in the effective population sizes of and significant genetic dilution in many South Indian cattle breeds such as the Vechur, Punganur, and Pulikulam breeds. However, the results of the present study indicate little or no critical loss of rare alleles in them. One of the plausible explanations for this scenario could be the potential gene flow from commercial taurine cattle through indiscriminate crossbreeding in the native tract of these breeds. Around 14.7%, 26.9%, and 22.2% of the sampled individuals of the Pulikulam, Vechur, and Punganur, respectively, had >12.5% taurine admixture [14]. Vechur and Pulikulam cattle have been reported to have a severe shortage of breeding males [23,24] in their respective native tracts. Relatively high artificial insemination coverage in these areas and access to purebred, commercial taurine cattle semen might have resulted in indiscriminate crossbreeding of purebred zebu cattle—thereby increasing the level of taurine admixture in them. The varying levels of genetic admixture resulting from crossbreeding with commercial taurine cattle might have increased the putative pre-bottleneck genetic diversity.

## 5. Conclusions

The present study revealed no concrete evidence for the occurrence of a recent genetic bottleneck in South Indian zebu cattle breeds. As bottlenecks tend to cause reductions in the genetic variability and fitness of populations, resources for the demographic monitoring of South Indian cattle need to be managed carefully and efficiently. Further initiatives need to be made for improving selective breeding practices in order to conserve and effectively utilize the existing South Indian zebu cattle genetic diversity. For example, the breed-specific conservation centres established by state governments may practice open nucleus breeding schemes not only to improve the stock of superior germplasm but also to improve diversity. Such centres may also serve as bull stations for the production and distribution of purebred semen for artificial insemination programs in native breed tracts. Furthermore, the awareness of farmers of data recording and scientific breeding practices needs to be ensured for the conservation and improvement of locally adapted cattle populations in South India.

## Figures and Tables

**Table 1:** Breed wise allelic diversity, mean expected HWE heterozygosity and mean expected mutation drift equilibrium heterozygosity under assumption of different models of microsatellite mutation.

Breed	*n*	ko	H_e_	H_eq_		
				IAM	TPM	SMM
Deoni	47	7.46	0.721	0.689	0.741	0.784
Hallikar	36	7.00	0.728	0.695	0.740	0.778
Kangayam	50	6.25	0.662	0.619	0.676	0.725
Ongole	49	6.75	0.679	0.651	0.707	0.754
Punganur	18	6.04	0.741	0.740	0.766	0.788
Pulikulam	34	8.08	0.740	0.753	0.792	0.823
Umbalachery	33	7.58	0.719	0.730	0.770	0.801
Vechur	26	6.04	0.672	0.679	0.718	0.751

He—Mean expected HWE heterozygosity; Heq—Mean expected mutation drift equilibrium heterozygosity.

**Table 2:** Sign test to evaluate zebu cattle breeds for mutation drift equilibrium under different models.

Breed	Infinite Alleles Model (IAM)				Stepwise Mutation Model (SMM)				Two Phase Model (TPM)			
	No. of loci with			*P*-Value	No. of loci with			*P*-Value	No. of loci with			*P*-Value
	H_e_ Deficit	H_e_ Excess			H_e_ Deficit	H_e_ Excess			H_e_ Deficit	H_e_ Excess		
		E	O			E	O			E	O	
Deoni	6	14.91	19	0.069	21	14.91	4	0.037	15	14.87	10	0.000
Hallikar	7	14.93	18	0.147	17	14.89	8	0.005	12	14.96	13	0.274
Kangayam	6	14.66	19	0.056	18	14.89	7	0.001	13	14.83	12	0.171
Ongole	8	14.79	17	0.245	20	14.85	5	0.000	14	14.87	11	0.086
Punganur	12	15.04	13	0.262	17	14.91	8	0.005	15	14.63	10	0.048
Pulikulam	15	14.96	10	0.036	20	14.78	5	0.000	17	15.02	8	0.004
Umblachery	10	15.02	15	0.572	15	14.91	10	0.037	13	15.05	12	0.149
Vechur	11	14.73	14	0.458	17	14.78	8	0.005	14	14.81	11	0.090

H_e_ = Heterozygosity, E = Expected, O = Observed.

**Table 3:** Standardized differences test to evaluate zebu cattle breeds for mutation drift equilibrium under different models.

Breed	Infinite Alleles Model (IAM)		Stepwise Mutation Model (SMM)		Two Phase Model (TPM)	
	T_2_	*P*-Value	T_2_	*P*-Value	T_2_	*P*-Value
Deoni	1.042	0.149	−8.147	0.000	−2.418	0.008
Hallikar	1.270	0.102	−5.911	0.000	−1.483	0.069
Kangayam	1.830	0.034	−5.038	0.000	−0.776	0.219
Ongole	0.366	0.357	−9.662	0.000	−3.282	0.000
Punganur	0.344	0.365	−4.375	0.000	−1.605	0.054
Pulikulam	−1.657	0.049	−13.109	0.000	−6.161	0.000
Umbalachery	−0.889	0.187	−9.881	0.000	−4.537	0.000
Vechur	−0.809	0.209	−8.130	0.000	−3.727	0.000

**Table 4:** Wilcoxon sign rank test to evaluate zebu cattle breeds for mutation drift equilibrium under different models.

Breed	Probability for One Tail Test (Heterozygosity Excess)		
	IAM	SMM	TPM
Deoni	0.015	1.000	0.831
Hallikar	0.100	0.998	0.729
Kangayam	0.024	0.996	0.625
Ongole	0.050	0.999	0.933
Punganur	0.213	0.998	0.909
Pulikulam	0.755	0.999	0.998
Umbalachery	0.604	0.993	0.943
Vechur	0.468	0.998	0.914

## Data Availability

The microsatellite dataset generated through this research work are stored in the DRYAD repository and the link to access the data is:https://datadryad.org/stash/share/TruWO74-F4wPcd0FnvHGBB0zEZ_supDxj0S2b3SZASw and the access date 1 June 2022.

## References

[1] Murray C., Huerta-Sanchez E., Casey F., Bradley D.G. (2010). Cattle demographic history modeled from autosomal sequence variation. Philos. Trans. R. Soc. Lond B Biol. Sci..

[2] Achilli A., Olivieri A., Pellecchia M., Uboldi C., Colli L., Al-Zahery N., Accetturo M., Pala M., Hooshiar Kashani B., Perego U.A. (2008). Mitochondrial genomes of extinct aurochs survive in domestic cattle. Curr. Biol..

[3] Loftus R., MacHugh D., Bradley D., Sharp P., Cunningham P. (1994). Evidence for two independent domestication of cattle. Proc. Natl. Acad. Sci. USA.

[4] Scherf B.D., Pilling D., FAO (2015). The Second Report on the State of the World’s Animal Genetic Resources for Food and Agriculture.

[5] Livestock Census: 20th Livestock Census of India-Key Results; Ministry of Fisheries, Animal Husbandry and Dairying, Department of Animal Husbandry and Dairying: Krishi Bhavan, India, 2019. https://epashuhaat.gov.in/documents/ProvisionalKeyResultsof20thLivestockCensus.pdf.

[6] ICAR-NBAGR (2016). Guidelines for Management of Animal Genetic Resources of India.

[7] Bradshaw C.J.A., Isagi Y., Kaneko S., Brook B.W., Bowman D.M.J.S., Frankham R. (2007). Low genetic diversity in the bottlenecked population of endangered non-native banteng in northern Australia. Mol. Ecol..

[8] Al-Atiyat R.M. (2008). Extinction probabilities of Jordan indigenous cattle using population viability analysis. Livest. Sci..

[9] Ganapathi P., Rajendran R., Kathiravan P. (2012). Detection of occurrence of a recent genetic bottleneck event in Indian hill cattle breed Bargur using microsatellite markers. Trop. Anim. Health Prod..

[10] Kataria R.S., Kathiravan P., Bulandi S.S., Pandey D., Mishra B.P. (2010). Microsatellite based genetic monitoring to detect cryptic demographic bottleneck in Indian riverine buffaloes (*Bubalus bubalis*). Trop. Anim. Health Prod..

[11] Glowatzki-Mullis M.-L., Muntwyler J., Bäumle E., Gaillard C. (2008). Genetic diversity measures of Swiss goat breeds as decision-making support for conservation policy. Small Rumin. Res..

[12] Amirinia C., Seyedabadi H., Banabazi M.H., Kamali M.A. (2007). Bottleneck study and genetic structure of Iranian Caspian horse population using microsatellites. Pak. J. Biol. Sci..

[13] Côté S.D., Dallas J.F., Marshall F., Irvine R.J., Langvatn R., Albon S.D. (2002). Microsatellite DNA evidence for genetic drift and philopatry in Svalbard reindeer. Mol. Ecol..

[14] Manomohan V., Saravanan R., Pichler R., Murali N., Sivakumar K., Sudhakar K., Raja K.N., Periasamy K. (2021). Legacy of draught cattle breeds of South India: Insights into population structure, genetic admixture and maternal origin. PLoS ONE.

[15] FAO (2011). Molecular Genetic Characterization of Animal Genetic Resources.

[16] Sambrook J., Russell D.W. (2001). Molecular Cloning: A Laboratory Manual.

[17] Grema M., Traoré A., Issa M., Hamani M., Abdou M., Soudré A., Sanou M., Pichler R., Tamboura H.H., Alhassane Y. (2017). Short tandem repeat (STR) based genetic diversity and relationship of indigenous Niger cattle. Arch. Anim. Breed..

[18] Gibbons J.D., Chakraborti S. (1992). Nonparametric Statistical Inference.

[19] Piry S., Luikart G., Cornuet J.M. (1999). BOTTLENECK: A computer program for detecting recent reductions in the effective population size using allele frequency data. J. Hered..

[20] Cornuet J.M., Luikart G. (1996). Description and power analysis of two tests for detecting recent population bottlenecks from allele frequency data. Genetics.

[21] Tantia M.S., Vijh R.K., Mishra B., Bharani Kumar S.T., Ahlawat S.P.S. (2006). Evaluation of Indian fowl populations for mutation drift equilibrium. Indian J. Anim. Sci..

[22] Kathiravan P., Mishra B.P., Kataria R.S., Sadana D.K. (2009). Evaluation of genetic architecture and mutation drift equilibrium of Marathwada buffalo population in Central India. Livest. Sci..

[23] DADF (2015). Estimated Livestock Population Breed-Wise Based on Breed Survey 2013.

[24] Singh P.K., Sharma A. (2017). Assessment of degree of endangerment of livestock breeds in India. Indian J. Anim. Sci..

[25] Di Rienzo A., Peterson A.C., Garza J.C., Valdes A.-M., Slatkin M., Freimer N.B. (1994). Mutational processes of simple sequence repeat loci in human populations. Proc. Natl. Acad. Sci. USA.

[26] Nei M., Chakraborty R., Fuerst P.A. (1976). Infinite allele model with varying mutation rate. Proc. Natl. Acad. Sci. USA.

[27] Garza J.C., Williamson E.G. (2001). Detection of reduction in population size using data from microsatellite loci. Mol. Ecol..

[28] Williamson-Natesan E.G. (2005). Comparison of methods for detecting bottlenecks from microsatellite loci. Conserv. Genet..

[29] Girod C., Vitalis R., Lebois R., Freville H. (2011). Inferring population decline and expansion from microsatellite data: A simulation-based evaluation of the MSvar methods. Genetics.

[30] Peery M.Z., Kirby R., Reid B.N., Stoelting R., Doucet-Beer E., Robinson S., Vasquez-Carrillo C., Pauli J.N., Palsboll P.J. (2012). Reliability of genetic bottleneck tests for detecting recent population declines. Mol. Ecol..

